# Energy Landscape Topography Reveals the Underlying Link Between Binding Specificity and Activity of Enzymes

**DOI:** 10.1038/srep27808

**Published:** 2016-06-14

**Authors:** Wen-Ting Chu, Jin Wang

**Affiliations:** 1 State Key Laboratory of Electroanalytical Chemistry, Changchun Institute of Applied Chemistry, Chinese Academy of Sciences, Changchun, Jilin, 130022, China; 2 Department of Chemistry & Physics, State University of New York at Stony Brook, Stony Brook, NY, 11794, USA.

## Abstract

Enzyme activity (often quantified by *k_cat_*/*K_m_*) is the main function of enzyme when it is active against the specific substrate. Higher or lower activities are highly desired for the design of novel enzyme and drug resistance. However, it is difficult to measure the activities of all possible variants and find the “hot-spot” within the limit of experimental time. In this study, we explore the underlying energy landscape of enzyme-substrate interactions and introduce the intrinsic specificity ratio (ISR), which reflects the landscape topography. By studying two concrete systems, we uncover the statistical correlation between the intrinsic specificity and the enzyme activity *k_cat_*/*K_m_*. This physics-based concept and method show that the energy landscape topography is valuable for understanding the relationship between enzyme specificity and activity. In addition, it can reveal the underlying mechanism of enzyme-substrate actions and has potential applications on enzyme design.

The enzyme activity defines the efficiency of an enzyme to convert its substrate into product^1^,^2^,^3^,^4^. In general, the catalytic activities of enzymes are reflected in the kinetic parameters *k*_*cat*_ and *K*_*m*_. The *k*_*cat*_ (often denoted as the turnover number) is the catalytic rate constant of the enzymatic reaction. It measures the amount of product formed per enzyme molecule per unit time. *K*_*m*_ (often denoted as dissociation constant) represents the binding affinity of the enzyme-bound substrate complexes^5^.

The Michaelis-Menten equation^6^ defines the rate *v* of product formation of an enzyme catalyzed reaction (at low [*S*]): 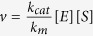. Comparison of the reaction rate between two different enzymes A and B that utilize the same substrate, transforms this equation into 
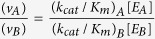
. Here, if the concentrations of the two enzymes are equal, the reaction rate is directly related to *k*_*cat*_/*K*_*m*_. Some enzymes can bind and catalyze various substrates. Conversely, one single substrate can be bound and catalyzed by different enzymes. However, *k*_*cat*_/*K*_*m*_ is specific for the catalytic efficiency of a given enzyme with a specific substrate. For this reason, *k*_*cat*_/*K*_*m*_ is known as the enzyme activity or “specificity constant”^1^.

Based on the free energy profile of the enzyme-substrate complex, it was speculated that^1^ the energy difference Δ*G* between the enzyme and substrate in the free state (*E* + *S*) and the enzyme-substrate complex in the transition state (*TS*) is linked to the activity *k*_*cat*_/*K*_*m*_ (illustrated in Fig. 1). As a result, this implies that *k*_*cat*_/*K*_*m*_ is at maximum when the enzyme is complementary to the transition state. The activity can be quantified by the complementarity of the transition state. However, at present this is still difficult to be measured in experiments and calculated in theory.

In practice, there is an urgent need to find the enzyme-substrate complex with the highest or lowest activity as fast as possible. In general, the conventional specificity measures the discrimination of the specific receptor against other receptors for a ligand binding (ΔΔ*G*_*bind*_ in free energy). In the previous studies^7^,^8^,^9^,^10^, we suggested that the conventional thermodynamic specificity of discriminating of a ligand with different receptors can be correlated to the intrinsic specificity of discriminating of a ligand with different binding sites (modes or conformations) of a large receptor (here the ligand is an inhibitor, not the substrate). The physical rational behind this connection is that exploring ligand interactions with the receptors through the sequences (different receptors) and exploring the ligand interactions with a receptor through conformations (different binding modes) are equivalent for large receptors. After comparison with the experimental data, it was illustrated in an example of Cox-2 inhibitor binding that, the intrinsic specificity quantified through exploring the underlying energy landscape is correlated with the experimentally measured conventional specificity. However, for enzymes, activity is related to both binding and catalyzing processes. Conventional specificity is not expected to correlate very well with enzyme activity.

Binding specificity is related to the change in interactions between the receptor and the ligand among a series of complexes. It can be quantified by intrinsic specificity based on the underlying binding energy landscape^7^,^11^ (Fig. 1, for the detailed theoretical basis, see section 1 of Supplementary). The shape of this binding energy landscape looks like a funnel towards the native binding state with local roughness along the binding paths^7^,^8^,^12^,^13^,^14^,^15^,^16^,^17^,^18^,^19^,^20^. The “native” conformation of the complex is the conformation with the lowest binding energy *E*_*n*_ (enzyme-substrate complex, *ES* state). Intrinsic specificity ratio, 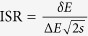, is defined to quantify the magnitude of intrinsic specificity, where *δE* is the energy gap between the energy of native conformation *E*_*n*_ and the average energy of all the conformational states; Δ*E* is the energy fluctuation or the width of the energy distribution of the non-native binding states, reflecting the roughness of the landscape^7^; S represents the configurational entropy. ISR is the ratio of the energy gap or slope of the landscape versus the roughness of the landscape modularized by the size of the system. Therefore, ISR reflects the topography of the underlying energy landscape. Large ISR shows more funneled binding landscape against local roughness and size of the system. Large ISR also implies discrimination of native state against non-native states, and therefore the high intrinsic specificity.

In our previous study, it has been suggested that intrinsical specificity is related to the conventional thermodynamic specificity of binding^7^,^8^,^9^,^10^. This correlation is established in protein-ligand systems, without a catalytic reaction taking place. In enzyme systems, the change in interactions between enzyme and substrate will not only influence the binding affinity, but also the kinetic rate of the enzymatic reaction. As a result, changes in *k*_*cat*_/*K*_*m*_ among a series of enzyme-substrate complexes (e.g. one substrate utilized by multiple different enzymes, or multiple different substrates utilized by one enzyme) are naturally linked to the changes in the interactions between enzyme and substrate. These changes in interactions will lead to the changes in the underlying energy landscape, which will be reflected in the ISR. Therefore, a link between ISR and enzymatic activity can be established. Recently, reaching the high activity is a grand goal for enzymology. Studies show that enzyme activity is often correlated to the stability of the enzyme^21^. Yet, it is still challenging to design highly active enzymes with high catalytic efficiencies, due to the fact that the designed enzymes usually have lower activity than the natural ones^22^,^23^. In this study, we will explore the relationship between the intrinsic binding specificity and enzyme activity, aiming to uncover the mechanism of enzyme activity from the underlying energy landscape topography. This will allow for a quantitative and reliable way of rapidly predicting and designing enzymes with high activities.

To begin our study, we chose substrate-enzyme system and compared the differences in activity between the wild-type and a series of variants of the enzyme (mutants). The serine proteases super-family is a perfect subject for studying of the enzyme specificity and activity^24^,^25^,^26^,^27^. In addition, another enzyme, *p*-hydroxybenzoate hydroxylase (PHBH)^28^,^29^,^30^,^31^ is also selected for comparison. In this study, we focused on the reaction mechanism and specificity of a single chymotrypsin-like serine protease, porcine pancreatic elastase (PPE)^32^,^33^,^34^ and PHBH systems. PPE requires several essential residues for catalysis, but PHBH accomplishes the catalytic reaction through only a co-factor. The substrate of PPE is a large long-chain molecule, whereas the substrate of PHBH is a small aromatic molecule. Both the binding and catalyzing processes of PPE and PHBH are different. Therefore, the study of the activity of these two systems is comprehensive and representative. Molecular dynamics (MD) simulations and the related quantum mechanical version (QM/MM) are useful methods for obtaining the chemical and physical characteristics of bio-molecules and their associated reactions. According to the transition state theory (TST)^35^,^36^,^37^,^38^,^39^, the reaction barrier can be measured to quantify the chemical kinetics. Because of the instability and uncertainty of the transition state, significant effort is required to calculate the reaction barrier. As a result, calculating properties of enzyme activity (i.e. *k*_*cat*_ and *K*_*m*_) with computational chemical methods, such as MD and QM/MM simulations, is very complicated and time-consuming. The correlation between ISR and enzymatic activity demonstrated in this study will allow one to rapidly obtain the valuable information of “hot-spot” of enzyme activity. This finding will be important for understanding the fundamental mechanism of enzymes as well as for predicting and designing new enzymes from the quantifications of the underlying landscape topography.

## Result

### Correlation between binding intrinsic specificity ratio and enzyme activity

**Figure 2:**
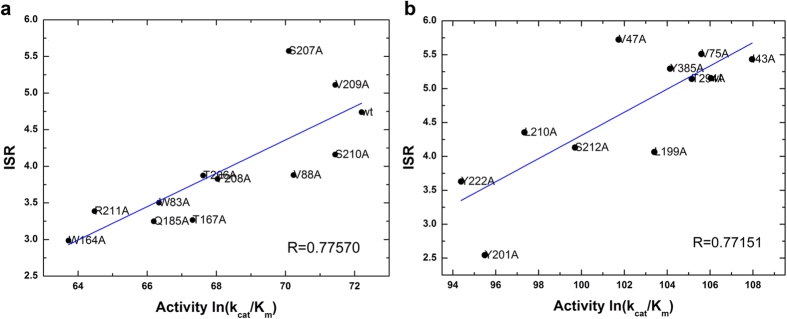
Connection of intrinsic specificity ratio (ISR) and enzyme-substrate activity (measured as *ln*(*k*_*cat*_/*K*_*m*_)) in PPE systems (**a**) and PHBH systems (**b**).

For PPE systems, the eleven mutation sites (W83A, V88A, W164A, T167A, Q185A, T206A, S207A, F208A, V209A, S210A, and R211A) were determined using MD simulations and MMGBSA calculations (see Section 3 of Supplementary). Once the docking pose distribution and the binding energy of each system were carried out, the intrinsic specificity ratio (ISR) of each system was calculated. The results of ISR of all the twelve systems are listed in Supplementary Table S1. The values of ISR range from 2.985 to 5.575. The correlation between ISR and enzyme-substrate activity (calculated through QM/MM simulations) is shown in Fig. 2a. There is an obvious positive correlation between them with *R* = 0.77570. ISR increases as activity increases. S207A, V209A, and wild-type systems have higher ISR and activity values; W164A, R211A, and Q185A systems have lower ISR and activity values. These results support the intimate correlation between ISR and activity when a given substrate binds to wild-type or mutated enzyme and catalysis occurs through a similar reaction mechanism.

For PHBH systems, ten mutation sites in the vicinity of *p*HB (I43A, V47A, V75A, L199A, Y201A, L210A, S212A, Y222A, T294A, and Y385A) were chosen for the activity and ISR calculations. ISR and its correlation with activity are shown in Supplementary Table S2 and Fig. 2b. Like PPE, there is a positive correlation between ISR and enzyme-substrate activity in PHBH systems, with *R* = 0.77151. The ISR values range from 2.544 to 5.723. Of all the mutations, I43A, V47A, V75A, T294A, and Y385A have ISR similar to the wild-type. And L199A, Y201A, L210A, S212A, and Y222A have ISR lower ISR than that of wild-type system. The correlation between ISR and activity in PHBH systems is consistent with that observed for PPE systems, which supports that ISR can imply the enzyme-substrate activity.

Significant efforts have been put forth to identify the enzyme-substrate pairs with the highest or lowest catalytic efficiency (or activity, *k*_*cat*_/*K*_*m*_) as a means of blocking drug toxicity or drug resistance. Pan *et al*.^40^ and Yang *et al*.^41^ applied QM/MM and free energy perturbation (FEP) simulations to theoretically evaluate the effect of mutations on the catalytic efficiency of human butyrylcholinesterase. It will be time-consuming to obtain the *k*_*cat*_ and *K*_*m*_ values of every possible mutation with experimental or theoretical approaches (the comparison between the computational time of normal MD and QM/MM methods and that of ISR method is shown in Supplementary Information). In general, the conventional specificity refers to the difference in binding affinity between wild-type and mutant enzymes. But for enzyme-substrate complex, the activity will also be affected by catalytical reaction. Two similar systems with the same binding affinity may have different activities. As a result, conventional specificity may not necessarily represent this difference in activity. For example, there is low correlation (*R* = 0.57358) between *ln*(*k*_*cat*_/*K*_*m*_) and affinity in the PPE system. Likewise, the activity is not directly related to the reaction barrier (*R* = 0.57532). Therefore, it is difficult to evaluate the changes in activity using conventional specificity (binding affinity) alone. ISR provides a more reliable method for comparing the efficiencies of different enzyme-substrate complexes.

In general, the ISR value reflects the receptor-ligand binding landscape topography. The relationship between ISR and binding affinity is clear; however the correlation between ISR and the catalytic barrier is not completely resolved. As we know, much lower activation energy is required to accomplish a reaction in enzyme than in aqueous solution. Residues in active site can facilitate the catalysis and help to stabilize the transition state, which will reduce the reaction barrier. And in same chemical reaction (with the same bond formed/broken), when the environment of enzyme is altered, the barrier will also be changed. That is to say, the environment of substrate is crucial for determining the reaction barrier. To our knowledge, for each enzyme-substrate pair, activity is a unique property. The interaction environment between enzyme and substrate determines the value of activity. As a result, the activities of the wild-type and mutated enzyme complexed with the same substrate can be compared based on the changes in interaction environment. When the critical catalytic residues are altered to others, the reaction mechanism may be different, or the reaction may not occur. But the change of other important residues in the environment (not directly involved in the reaction) will have the same reaction mechanism with a bit higher or lower reaction barrier. This is because the mutation may change the interaction environment between the enzyme and the substrate, as well as the interaction environment between the enzyme and the transition state (TS) form of the substrate. Moreover, the enzyme-substrate binding landscape not only reflects the interaction within the native enzyme-substrate complex, but also the statistics of the non-native enzyme-substrate complexes. Both energy gap and roughness of the funnel-like binding energy landscape reflect the ensemble of enzyme-substrate complexes and provide information on the nature of interaction environment. Our results support that the mutants can influence the gap and roughness of the binding energy landscape. ISR, in the form of ratio of energy gap and roughness, shows the trends of discrimination of interaction environment through a series of enzyme-substrate complexes. Consequently, the ISR can directly reflect the relative value of enzyme activity or specificity.

### The relationship among ISR, conventional specificity, and kinetic specificity

As we know, the activity of enzyme-substrate complex is related to both binding affinity (*K*_*m*_) and catalytic reaction rate (*k*_*cat*_). In our previous study, it was demonstrated that thermodynamic intrinsic specificity correlates with conventional specificity and kinetic specificity^9^,^10^. In these studies of the receptor-ligand complex, the conventional specificity is measured as the difference between the affinities of the same ligand binding with different receptors; and the kinetic specificity is measured as the kinetic residence time of each ligand. In this study, we used the same substrate with the different enzymes, therefore we define the conventional specificity as binding energy (the relative value of which is the same as using the difference between affinities of the substrate binding to wild-type and mutant enzymes). For each complex, the substrate firstly binds to enzyme, then catalysis takes place. The rate-limited step of all the kinetic processes is the catalytic step (in PPE, second step of catalytic reaction, acylation reaction is the rate-limited step). Therefore, the reaction barrier of catalytic reaction is measured as the kinetic specificity.

**Figure 3:**
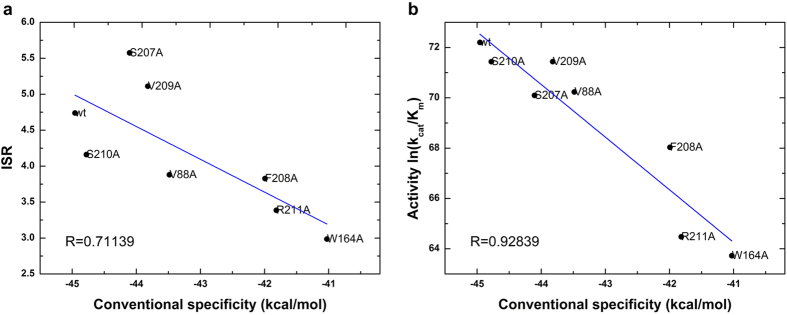
Connection of (**a**) intrinsic specificity ratio (ISR) and conventional specificity (measured as binding affinity), (**b**) activity (*ln*(*k*_*cat*_/*K*_*m*_)) and conventional specificity in PPE systems. The systems with reaction barrier within 1.5 kcal/mol of wild-type system are collected for statistics.

**Figure 4:**
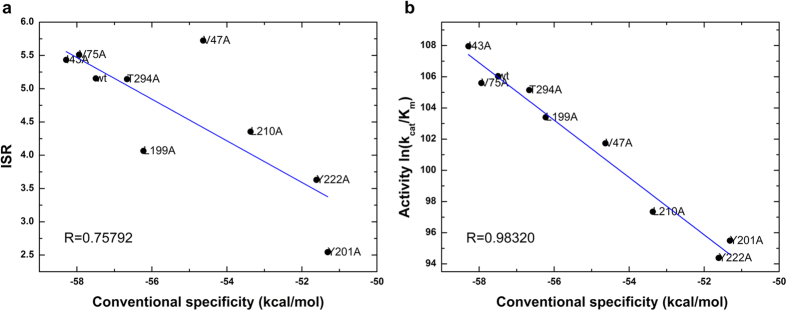
Connection of (**a**) intrinsic specificity ratio (ISR) and conventional specificity (measured as binding affinity), (**b**) activity (*ln*(*k*_*cat*_/*K*_*m*_)) and conventional specificity in PHBH systems. The systems with reaction barrier within 1.5 kcal/mol of wild-type system are collected for statistics.

The ISR value has a low correlation with conventional specificity (binding affinity difference) directly. However, if multiple systems with similar reaction rate (reaction barrier) are analyzed together, there is an obvious correlation between ISR and conventional specificity. As shown in Figs 3a and 4a, the systems with significantly different reaction barrier between wild-type (1.5 kcal/mol higher or lower) were not considered for the analysis. The activity is determined by both affinity and catalytic reaction rate. Likewise, if we remove the variants of reaction rate, the activity of these systems correlates with the conventional specificity. These results show that ISR correlates with the conventional specificity for the enzyme-substrate complexes that have the same reaction rate.

**Figure 5:**
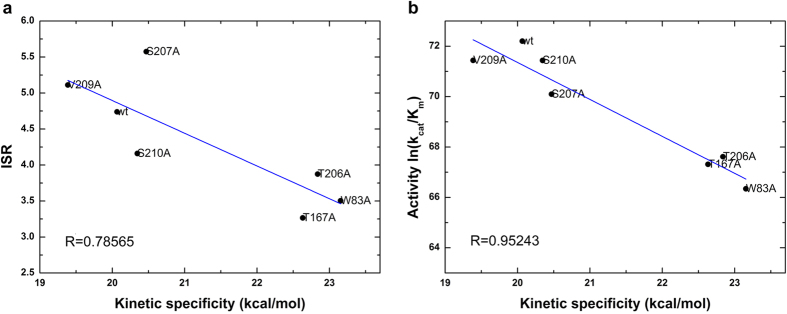
Connection of (**a**) intrinsic specificity ratio (ISR) and kinetic specificity (measured as reaction barrier), (**b**) activity (*ln*(*k*_*cat*_/*K*_*m*_)) and kinetic specificity in PPE systems. The systems with binding affinity within 1.5 kcal/mol of wild-type system are collected for statistics.

Similarly, ISR has a low correlation with kinetic specificity (reaction barrier) directly. Analysis of PPE mutated systems with binding affinity comparable to the wild-type system showed that ISR has correlation with the kinetic specificity (reaction barrier) at similar binding affinity level (Fig. 5a). In PHBH systems, reaction barriers of mutations are almost similar as that of wild-type PHBH. Therefore, the activity of PHBH shows low correlation with the reaction barrier. There were few mutations of PHBH with similar binding affinities but different reaction barriers compared with wild-type. In summary, in addition to enzyme activity, the ISR also has correlations with conventional specificity and kinetic specificity, in the case of similar catalytic reaction rate and similar binding affinity, respectively.

Intriguingly, there is no correlation between binding affinity and reaction barrier. That is to say, the level of substrate binding affinity can not influence the level of the rate of the catalytic process directly. But for the systems with similar activity, when the binding affinity increases (lower binding energy), the reaction rate decreases (higher reaction barrier). These results are consistent with the transition state theory.

### Residual type and location

**Figure 6:**
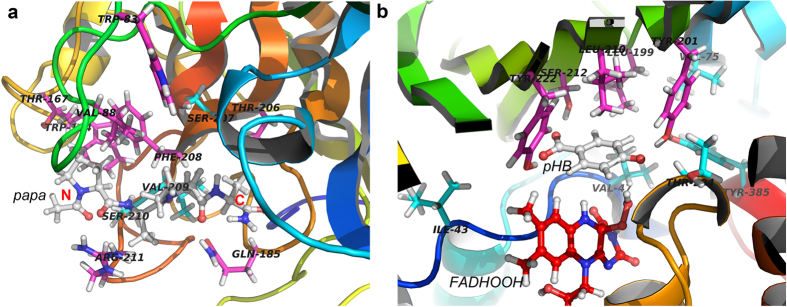
The locations of all the mutation sites and “hot spots” in PPE (**a**) and PHBH (**b**) systems. Hot spots are shown in magenta sticks, other mutation sites in cyan. The substrate and co-factor are illustrated in white and red ball-and-sticks, respectively. The N- and C-terminal of substrate PAPA of PPE are labeled in red.

Of all the twelve systems (wild-type and eleven mutants) of PPE, there were eight systems with ISR values lower than 4.0 (W83A, V88A, W164A, T167A, Q185A, T206A, F208A, and R211A), which are classified as lower specificity systems. These residues Tyr83, Val88, Tyr164, Thr167, Gln185, Thr206, Phe208, and Arg211 are referred to as “hot spots” due to the change in enzymatic activity when they are altered (shown in Fig. 6a). In three lower specificity systems (W83A, W164A, and F208A), the side chain of the amino acids are changed from a large aromatic ring (wild-type) to the −CH_3_ group (mutant). The ISR values of these systems are 3.503, 2.985, and 3.827, respectively. These mutants have a much lower energy gap (*δE*) than wild-type, which means that these systems have less biased or less funneled energy landscapes towards the native binding complex. Moreover, R211A, which has its long charged side chain replaced with a −CH_3_ group, also has a much lower ISR value (3.386). These results indicate that in PPE, the side chain of the residues in active site is crucial for the ISR and enzymatic activity. When they are mutated from large aromatic or long charged side chain to Ala, the activity of these systems will be greatly influenced.

In the enzyme PPE, the substrate is mostly surrounded by loop regions, and almost all of the mutation sites in our study are located in these loop regions. As shown in Supplementary Fig. S6, the residues with high contributions to binding belong to five important loop regions in the active site of PPE. They are loops around His45, Val88, Trp164, Gln185-Asp187, and Ser207-Arg211. Residues within the loops were selected for building the mutation systems, with the exception of His45, which is known to be a member of the enzyme’s catalytic triad. The eleven mutants can be divided into four groups based on their locations within the loop regions. Group 1, Trp83 and Val88; Group 2, Trp164 and Thr167; Group 3, Gln185; Group 4, Thr206, Ser207, Phe208, Val209, Ser210, and Arg211. In complexed structure of PPE, Group 1 and Group 2 are located near the first amino acid at N-terminal of substrate (Pro- in peptide substrate PAPA). The side chain of Phe208 and Arg211 of Group 4 are also near to the first amino acid in N-terminal of PAPA, but Ser207, Val209, and Ser210 are in close proximity to the two middle residues of PAPA. Phe208 and Arg211 have strong van der Waals interactions with the first Pro residue. Ser207, Val209, and Ser210 form hydrogen bonds with the backbone atoms of PAPA. The Gln185 of Group 3 and the Thr206 of Group 4 are located around the C-terminal of PAPA. In summary, the mutations of residues located near the N-terminal and C-terminal of substrate PAPA have lower ISR and activity values, whereas mutations in close contact with the middle two residues of PAPA have similar ISR and activity as the wild-type system (see Fig. 6a). This suggests that in PPE, the residues that have interactions with the head and tail of substrate will have an effect on the specificity of enzyme-substrate complex.

In PHBH systems, the situation is simpler than the PPE systems. There are five lower specificity systems with ISR lower than 4.5. They are L199A, Y201A, L210A, S212A, and Y222A. These hot spots are all located in the opposite direction of co-factor FADHOOH with respect to substrate *p*HB (shown in Fig. 6b). Therefore, residues in this region are essential for the activity of PHBH. In addition, in wild-type PHBH, Tyr201, Ser212, and Tyr222 form direct hydrogen bonds with *p*HB. They have important contributions for the binding affinity. Altering these residues results in a much lower energy gap (*δE*) than the wild-type, which as described for PPE systems, means that these systems have less biased or less funneled energy landscapes towards the native binding complex, with less discrimination or intrinsic specificity, and therefore less activity.

## Discussion

We analyzed two different systems, chymotrypsin-like serine protease, PPE (substrate PAPA) and *p*-hydroxybenzoate hydroxylase, PHBH (substrate *p*HB), total of 21 different single-point mutations of the perspective enzyme as a means of elucidating the relationship between intrinsic specificity and enzyme activity. Both the binding and catalyzing processes of PPE and PHBH are different. Therefore, studying the activity of these two systems is comprehensive and representative. The detailed two-step reaction mechanism of the acylation reaction of PPE, and one-step OH-transfer reaction mechanism of PHBH were provided through the QM/MM molecular dynamic simulations. The binding affinity, reaction barrier, and ISR of each complex were quantified by using molecular docking and molecular dynamics simulations. The results from our study show a statistical correlation between intrinsic specificity ratio (ISR) and enzyme activity. Additionally, the ISR correlates with the conventional specificity when reaction barriers are similar; and it correlates with kinetic specificity when binding affinities are similar. Our studies also suggest the “hot spots” (Tyr83, Val88, Tyr164, Thr167, Gln185, Thr206, Phe208, and Arg211 for PPE system; L199A, Y201A, L210A, S212A, and Y222A for PHBH system) located within an enzyme’s active site for determining the enzymatic activity.

Determining ISR to evaluate the enzyme activity has several advantages. First, this method is more time efficient than conventional computational simulations and wet lab experiments. ISR makes it possible to evaluate the activity of large enzymes, while the affinity and catalytic barrier of which are difficult to be computed in longtime-MD simulations (normal MD simulations, QM/MM MD simulations, and free energy calculations) at the current stage. Furthermore, the detailed mechanistic steps of the reaction being analyzed do not have to be fully understood prior to ISR determinations. Many catalytic reactions of enzymes are too complicated to study completely with experimental or computational approaches, therefore, ISR can be calculated quickly to predict the trends of enzymatic activity. In this sense, ISR has the predictive power for engineering and design for important enzyme activity. However, the method of ISR has some limitations. On the one hand, the reaction mechanisms (the broken and formed bonds) are assumed be similar for wild-type and mutated enzyme. This assumption ensures that the changes in enzymatic activities are solely due to the changes in the interaction environment between enzyme and its substrate. On the other hand, some mutations outside the active site can also influence the activity. In general, these mutations change the interaction environment through the net of hydrogen bonds and hydrophobic interactions (indirect interaction environment). As a result, an efficient equilibration step of the conformation of these mutations should be performed before docking. Through the fast analysis of activity with ISR calculations, one can change the activity of enzyme by altering one or more amino acids of the enzyme. This process will greatly aid in designing new types of enzymes and reducing the drug resistance based on the intrinsic specificity.

## Methods

### Initial models of systems

Atomic coordinates and structure factors for the protein PPE (240 a.a.) were built from the crystal structure 1HAZ^34^ (PPE and human beta-casomorphin-7 complex). The crystal structure 1IUW^42^ (PHBH complexed with FAD and *p*HB) was selected for constructing the model of PHBH system. The FAD in this structure was replaced by the oxidized form FADHOOH. After removing the ligand, the remaining fragment of the protein was selected as the initial structure for MD simulation. All water molecules within the crystal structure were retained in the initial model for simulations. The protonation states of all the residues of PPE were carefully defined according to the on-line toolkit PROPKA^43^ (http://propka.ki.ku.dk/). For PPE, residue His45 was single-protonated at the *δ* site and Asp93 was deprotonated according to the proposed mechanism. All missing hydrogen atoms of PPE were added using the LEaP module in the AMBER 12 package^44^. The ff99SB force field^45^ was applied to produce the parameters for the protein.

Once the residues with high contribution to the substrate binding were gained, eleven mutants of wild-type PPE-PAPA complex were chosen. The binding site of PHBH is rather smaller than PPE. Therefore, only ten residues in the vicinity of the substrate *p*HB were selected for mutation sites. The general Amber force field (GAFF)^46^ was used to obtain the force field parameters for the ligand and co-factor. Finally, an appropriate number of counterions were placed in each system, which was then solvated in an octahedral periodic box of TIP3P water molecules with a minimum distance of 8.0 Å between the outermost protein atoms and the walls of the simulation box. After MD simulations of each mutated complex, alanine scanning method was applied to obtain their binding free energies (for detail, see sections 2.2 and 3 of Supplementary). When all the wild-type and mutated protein-substrate complex were obtained, QM/MM MD simulations were performed to calculate the reaction barrier of each complex (for detail, see section 2.3 and 4 of Supplementary). Once the binding constant (*K*_*m*_) and reaction barrier (*k*_*cat*_) were calculated, the activity (*k*_*cat*_/*K*_*m*_) was readily obtained.

### Molecular docking and ISR calculation

Initially, the ligands PAPA and *p*HB were docked into wild-type and mutated forms of PPE and PHBH by AutoDock 4.2^47^, respectively, aiming to obtain the stable enzyme-substrate complexes for catalytic reaction. The boxes of docking were set to 70 × 70 × 70 grids and 85 × 85 × 85 grids, respectively, with 0.375 of grid spacing. The box size was large enough to cover the active site as well as the surrounding surface. One of the particle swarm optimization (PSO)-based optimizers^48^,^49^,^50^,^51^,^52^, FIPSDock^52^, was implemented into the protein-ligand docking program AutoDock. Generally, PSO-based algorithms show superior performance than the EA-based algorithms, especially for highly flexible ligands^53^. In conformational searching, the receptors PPE and PHBH-FADHOOH were considered to be rigid and the ligands PAPA and *p*HB were considered to be flexible with their torsion bonds defined by AutoDock according to their chemical features. The maximum number of energy evaluations was set as large as 2,500,000 to obtain the most stable conformation of PPE-PAPA and PHBH-*p*HB complexes.

After the construction and MD simulations of the mutated PPE and PHBH proteins, an equilibrated structure was obtained for each system. An ensemble of 1000 decoy conformations for each equilibrated structure (wild-type and mutants) were sampled, using FIPSDock as sampling method. The maximum number of energy evaluations was set to 250,000 to generate a sufficient sampling of possible docking poses for the binding sites of PPE and PHBH. Once the docking ensemble was obtained, a short MD run was performed on each docking complex, and MMGBSA was applied to calculate binding energy of them. The decoy with the lowest binding energy in each complex was considered as the “native” conformation. After that, an intrinsic specificity ratio (ISR) for each system would be calculated. Here, considering the ligand of each system is identical, the change of ligand entropy was ignored. The ISR in this manuscript is calculated as (*δE*)/(Δ*E*), which is unit-less.

## Additional Information

**How to cite this article**: Chu, W.-T. and Wang, J. Energy Landscape Topography Reveals the Underlying Link Between Binding Specificity and Activity of Enzymes. *Sci. Rep.* **6**, 27808; doi: 10.1038/srep27808 (2016).

## Supplementary Material

Supplementary Information

## Figures and Tables

**Figure 1:**
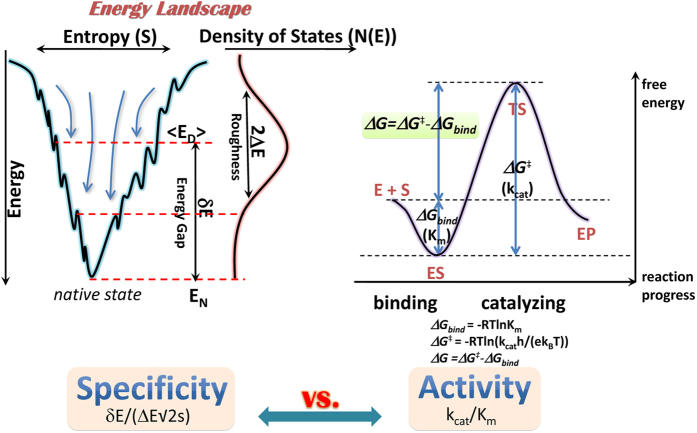
The definition of ISR (left) and the free energy profile of binding and catalyzing processes of the enzyme-substrate system (right). ISR is calculated by the roughness (Δ*E*) of distribution of binding energies, the energy gap or slope (*δE*) between the energy of native state (*E*_*n*_) and average energy (〈*E*_*D*_〉), and size measured by the entropy (S). ISR reflects the underlying landscape topography and quantifies the degree of discrimination of native state against decoys. And the energy landscape of binding is a funneled shape towards the native state. The activity (*k*_*cat*_/*K*_*m*_) can be transformed to the free energy difference (Δ*G*) between catalytic reaction barrier (Δ*G*^‡^) and binding free energy (Δ*G*_*bind*_). In this study, it was suggested that there is a significant statistical correlation between ISR values and activities.
