# Supplementary material for: Energy Landscape Topography Reveals the Underlying Link Between Binding Specificity and Activity of Enzymes
Source: Sci Rep. 2016 Jun 14;6:27808. doi: 10.1038/srep27808 (PMC4906287; doi:10.1038/srep27808)
Supplement: Supplementary Information [file srep27808-s1.pdf]

# Supplementary Information:

# Energy Landscape Topography Reveals the Underlying Link Between Binding Specificity and Activity of Enzymes

**Wen-Ting Chu,**[1] **and Jin Wang,**[1,2*]

[1] *State Key Laboratory of Electroanalytical Chemistry, Changchun Institute of Applied Chemistry, Chinese Academy of Sciences, Changchun, Jilin, 130022, China*
[2] *Department of Chemistry & Physics, State University of New York at Stony Brook, Stony Brook, NY, 11790, USA*

[*] *Correspondence to*:
jin.wang.1@stonybrook.edu (Jin Wang)

# 1 Theory

## 1.1 Intrinsic specificity ratio

The process of protein-ligand binding can be physically quantified and visualized as a funnel-like energy landscape towards the native binding state with local roughness along the binding paths.[1–10] The "native" conformation of the complex is the conformation with the lowest binding energy $E_n$. The energies of the all the non-native conformations follow a statistical Gaussian-like distribution[1,11](Figure 1). According to the energy landscape of binding[1,2,10,12–16], we can describe the processes of an enzyme binds a substrate with the reaction coordinate of binding.

The total Hamiltonian energy function of a protein-ligand complex can be expressed as

$$H = -\sum_{i,j} \varepsilon_{ij} \sigma_{ij} \tag{S1}$$

where $\varepsilon_{ij}$ representing the interaction strength between an atom $i$ of the protein and atom $j$ of the ligand is assumed to be random variable following a Gaussian distribution, which is related to the random energy model[17]. And $\sigma_{ij}$ equals 1 when the distance of the atoms $i$ and $j$ between protein and ligand is within the cutoff distance $R_c$ (about $3 \sim 5 \AA$ for common protein-ligand complex); $\sigma_{ij}$ equals 0 otherwise (unbound). Based on the statistical energy landscape theory[14,18], the native state $n$ has the lowest energy $E_n$, which is located at the bottom of the funnelled energy landscape. The native state is set as the enzyme-substrate complex (uncatalyzed state, native binding state, $ES$), as shown in Figure 1. Other than the native state, there are non-native binding states. In general, mutants of catalytic residues will lead to very low or none catalytic efficiency. Therefore, the mutants of other non-catalytic residual sites are selected in our studies. We only care about the free energy change between different systems. The energy of the catalytic residues almost does not change when compared among these mutants. The change of reaction barrier mostly comes from the change of environment, mainly around the mutated sites.

If we denote one $\sigma_{ij} = 1$ as a native interaction, just as the previous studies[1,2,19], for a conformation $a$ with energy $E_a$ and an overlap $Q$ with native state $n$, the fraction

of native contact interactions of state $a$ is

$$Q = \frac{1}{N} \sum_{i,j} \sigma_{ij}^{a} \sigma_{ij}^{n} \tag{S2}$$

where $N$ is the total number of native contact interactions. $Q$ is similar to the definition of reaction coordinate. By averaging over Gaussian distributions of interaction energy $\varepsilon_{ij}$, the probability from equation Eq. S1 is given by

$$\frac{P_{an}(E_a, Q, E_n)}{P_n(E_n)} = \frac{\left\langle \delta\left[E_a - H\left(\left\{\sigma_{ij}^{a}\right\}\right)\right] \delta\left[E_n - H\left(\left\{\sigma_{ij}^{n}\right\}\right)\right]\right\rangle}{\left\langle \delta\left[E_n - H\left(\left\{\sigma_{ij}^{n}\right\}\right)\right]\right\rangle} \tag{S3}$$

Eq. S3 can be also written in terms of Q

$$\frac{P_{an}(E_a, Q, E_n)}{P_n(E_n)} \sim \exp\left(-\frac{\left[(E_a - \bar{E}) - Q^{m-1}(E_n - \bar{E})\right]^2}{2N\Delta\varepsilon^2\left(1 - Q^{2(m-1)}\right)}\right) \tag{S4}$$

where $m$ is the order (multi-body interactions) of interactions between protein and ligand, $\bar{E}$ is the mean energy, and $\Delta\varepsilon^2$ is effective width or spread of the energy distribution per interaction.

In the microcanonical ensemble description of the thermodynamics, the energy and entropy of system are obtained as

$$E(T, Q, E_n) = \bar{E} + Q^{m-1}(E_n - \bar{E}) - \frac{N\Delta\varepsilon^2\left(1 - Q^{2(m-1)}\right)}{T} \tag{S5}$$

$$S(T, Q, E_n) = N s_{tot}(Q) - \frac{N\Delta\varepsilon^2\left(1 - Q^{2(m-1)}\right)}{2T^2} \tag{S6}$$

The conformational entropy $S_{tot}(Q)$ has relationship with $s_{tot}(Q)$ as $s_{tot}(Q) = S_{tot}(Q)/N$. We define that $\delta E$ is the energy gap between the energy of native conformation $E_n$ and the average energy of all the conformational states $\bar{E}$, $|E_n - \bar{E}|$, reflecting the slope of the landscape; and $\Delta E$ is the energy fluctuation or the width of the energy distribution of the decoys, reflecting the roughness of the landscape[1]. $\delta\varepsilon_n$ equals to $\delta E/N$, which is the energy gap per contact interaction; $\Delta\varepsilon^2$ equals to $\Delta E^2/N$, which represents the effective width of the energy distribution per contact interac-

tion. Trapping temperature $T_g$, at which the system is trapped with zero entropy $S(T,Q,E_n) = 0$, becomes $\Delta\varepsilon\sqrt{\frac{1}{2s_{tot}(Q=0)}}$ when $Q = 0$.

As illustrated in Figure 1, in binding process between enzyme and substrate, the minimum of $Q \sim 1$ represents the native binding state, and $Q \sim 0$ towards free energy minimum of unbound states. The free energy is given as: $F = E - TS$. Therefore, the transition temperature $T_t$ between the completely unbound states and native binding state is determined by the equality of the free energy minima at the completely unbound states and the free energy minima at native binding state ,which can be given by

$$T_t = \frac{\delta\varepsilon_n}{2s_{tot}\,(Q=0)} \times \left(1 + \sqrt{1 - \frac{2s_{tot}\,(Q=0)\,\Delta\varepsilon^2}{\delta\varepsilon_n^2}}\right) \tag{S7}$$

When taking the ratio of $T_t$ and $T_g(Q=0)$, we obtain

$$T_t/T_g\,(Q=0) = \Lambda + \sqrt{\Lambda^2 + 1} \tag{S8}$$

where $\Lambda = \frac{\delta\varepsilon_n}{\Delta\varepsilon\sqrt{2s_{tot}(Q=0)}}$, which represents the property of energy landscape of enzyme-substrate system. Large $\Lambda$ implies a funneled landscape against roughness and conformational states. Here we denote $\Lambda$ as the intrinsic specificity ratio (ISR)

$$ISR = \frac{\delta E}{\Delta E\sqrt{2S}} \tag{S9}$$

where $\sqrt{2S}$ is the scaling factor which accounts for the contribution of the entropy ($S_{tot}\,(Q)$) to the specificity or the size of the landscape or the system. Large ISR implies discrimination of native state against non-native states, and therefore the high intrinsic specificity.

By docking studies from substrate to enzyme, the ISR value can be obtained by Eq. S9. $\delta E$ and $\Delta E$ can be calculated by

$$\delta E = |E_n - \langle E_D \rangle| \tag{S10}$$

$$\Delta E = \sqrt{\langle E_D^2 \rangle - \langle E_D \rangle^2} \tag{S11}$$

where $E_D$ is the binding energy of each docking configuration (decoy). $\langle\rangle$ means the

average over the ensemble of decoys. Therefore, ISR quantifies the discrimination of native binding mode against the nonnative binding modes modularized by the size of the system. ISR also reflects the shape of the topography of the underlying landscape by providing the relative measure of the slope of the landscape biasing towards native state against the roughness of the landscape modularized by the size of the landscape. As a result, ISR is a direct measure of landscape topography including information on landscape slope, roughness and size.

### 1.2 Relationship between ISR and the activities of enzyme

In summary, intrinsic specificity ratio (ISR) correlates with conventional specificity when ligand binding to protein. In enzymes, the change of the interactions between enzyme and substrate caused by mutations will influence the topography of binding energy landscape, which can be easily quantified by ISR. As a result, the ISR can be used to reflect the trends of activity $k_{cat}/K_m$ among a series of enzyme-substrate complexes. Moreover, enzyme activity may not necessarily correlate with conventional specificity (binding affinity difference) or kinetic specificity (catalytic rate) alone. But when conventional specificity or kinetic specificity is fixed, the change of activity should be consistent with the other characteristic. So as to ISR, according to the properties of binding energy landscape, we expect ISR to include both the characteristics of conventional specificity and kinetic specificity (as shown in Fig. S1). After accurate simulations with different methods, we will show that ISR has relationship with the important properties of enzyme activity, conventional specificity, and kinetic specificity. Therefore, ISR is very useful and informative.

## 2 Materials and methods

### 2.1 Molecular dynamics simulation

Firstly, the isolated PPE and PHBH-FADHOOH were prepared for classical MD simulations. Two steps of energy minimizations were performed. All the water molecules and counterions were minimized with 5000 steps of steepest descent followed by 5000 steps of conjugate gradient. Then the whole system was minimized

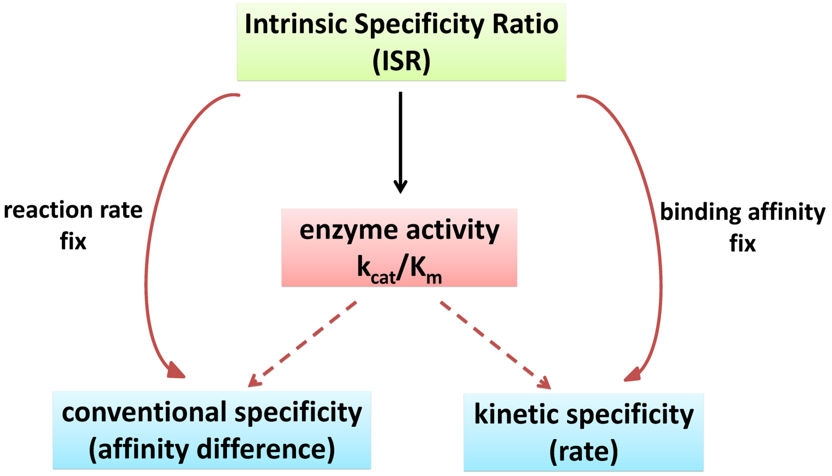


**Fig. S1** The relationship between ISR and the important properties of enzyme, activity, conventional specificity, and kinetic specificity. Straight line means there is a direct correlation between the two quantities; dashed line means no direct correlation; curve means correlation under some conditions.

with the same process to remove the bad contacts. The system was gradually heated up to 300 K in the NVT ensemble, with velocity of 1 K/ps. It reached equilibration after another 200 ps simulation. In the meantime, weak restrains were performed on the C$\alpha$ atoms of the protein during the first two processes to ensure the accomplishment of the stabilization. Finally, 5 ns MD simulation was carried out with the NPT ensemble. A SHAKE algorithm[20] was applied to constrain all bonds involving hydrogen atoms. Particle mesh Ewald (PME) method[21] was used to calculate the electrostatic interactions with a cutoff value of 12 Å. The time step was set to 2 fs.

After docking, all the 12 complex systems of PPE and 11 complex systems of PHBH were prepared for 2 ns MD simulation. The pre-treatment and all the settings of MD simulation of each system were the same as that of iso-protein.

## 2.2 MMGBSA calculation

After product MD simulation, the binding free energy of each complex was calculated through the MMGBSA method.[22,23] In MMGBSA, the binding free energy, $\Delta G_{bind}$, is written as the sum of molecular mechanics energy, $\Delta E_{MM}$, salvation free energy contribution to binding, $\Delta G_{sol}$, and the conformational of entropic contribu-

tion, $-T\Delta S$. This can be represented as in Eq. S12:

$$\Delta G_{bind} = \Delta E_{MM} + \Delta G_{sol} - T\Delta S \tag{S12}$$

where both $\Delta E_{MM}$ and $\Delta G_{sol}$ can be divided into two parts:

$$\Delta E_{MM} = \Delta E_{ele} + \Delta E_{vdw} \tag{S13}$$

$$\Delta G_{sol} = \Delta G_{gb} + \Delta E_{np} \tag{S14}$$

The $\Delta E_{ele}$ and $\Delta E_{vdw}$ in Eq. S13 are the electrostatic interaction and van der Waals energy in the gas phase, the $\Delta G_{gb}$ and $\Delta G_{np}$ in Eq. S14 are the polar and nonpolar contributions to the solvation free energy, respectively. The polar contribution of the solvation energy $\Delta G_{gb}$ was calculated using the generalized Born model[24]. The nonpolar contribution of salvation energy $\Delta G_{np}$ was calculated by Eq. S15:

$$\Delta G_{np} = \gamma SASA + \beta \tag{S15}$$

The solvent accessible surface area (SASA) in Eq. S14 was estimated using ICOSA model in AMBER. In this study, the values for $\gamma$ and $\beta$ were set as default values, 0.0072 kcal/(mol·Å$^2$) and 0 kcal/mol.

For each system, 950 snapshots were extracted from the 2 ns MD trajectory of the complex at an interval of 1 ps (first 50 ps is not included for energy calculation). Because there was just one mutation site of each mutated system, the difference of entropy between the mutated system and wild-type system was rather small. As we only care about the relative value of energy but not the absolute value, here we did not calculate the entropy of each system. For wild-type PPE and PAPA complex, per-residue energy decomposition was also performed on the wild-type complex to determine the mutation sites. However, because of the small size of substrate *p*HB, the binding site of PHBH is rather smaller than PPE. For sampling the mutant systems as much as possible, we select the residues in the vicinity of substrate *p*HB for mutation sites. After the MD simulation of mutated systems, alanine scanning method was used to obtain the binding energy of each mutated system.

## 2.3 QM/MM simulation

The proposed reaction mechanisms of the acylation step of amidase activity of PPE and the OH-transfer step of PHBH are illustrated in Fig. S2 and Fig. S3. A QM/MM MD simulation was implemented on the equilibrated complex structure of each system to calculate the mechanism of the acylation reaction of amidase activity. And the umbrella sampling (US)[25,26] method was applied on each system to enhance the sampling of the transition states. Umbrella sampling simulation can drive the enzyme system over the energy barrier by harmonically restraining the reaction coordinate (RC) to a series of windows. For PPE systems, the QM part of the MD simulations included part of the substrate PAPA, residue His45, and Ser188. In PHBH systems, the QM part was composed of substrate *p*HB and part of flavin group of FADHOOH. For accuracy, we cut the C−C single bond between C13 and C14, CA and CB, C12 and C14. Hydrogen link atoms were introduced along the covalent bonds crossing the boundary between the QM and the MM regions, to satisfy the valence requirements of the QM fragments. In PPE systems, the QM region has 34 atoms (included 3 hydrogen link atoms, as shown in Fig. S4C). In PHBH systems, the QM region has 49 atoms (included 1 hydrogen link atoms, as shown in Fig. S5C). The rest of the complex system was the MM part. SCC-DFTB semi-empirical method[27,28] and ff99SB force field were applied on each part of the system. For the first step of reaction of PPE systems, the length of *R(HG-OG)-R(NE2-HG)-R(OG-C17)* (denoted as d2-d1-d3) was set as RC1 ($\xi_1$); for the second step, *R(NE2-HG)+R(N4-C17)-R(HG-N4)* (denoted as d1+d5-d4) was set as RC2 ($\xi_2$), as illustrated in Fig. S4C. In PHBH systems, the length of *R(O86-O87)-R(O87-C6)* (denoted as d1-d2) was set as RC3 ($\xi_3$), as depicted in Fig. S5C). RC1, RC2, and RC3 were ranging from about -4.2 to 1.4 Å, from about 0.5 to 5.0 Å, and from -1.7 to 1.7 Å, respectively. As a result, 57 windows, 56 windows, and 35 windows were sampled along these RCs in step of 0.1 Å, respectively. For each window, 5 ps of equilibration and 20 ps of US sampling were performed with a force constant of 100.0 kcal/(mol·Å$^2$). Each subsequent simulation was started from the last frame of the previous run. After getting the distribution of the reaction coordinate of each window, the weighted histogram analysis method (WHAM)[25,29] was used to obtain

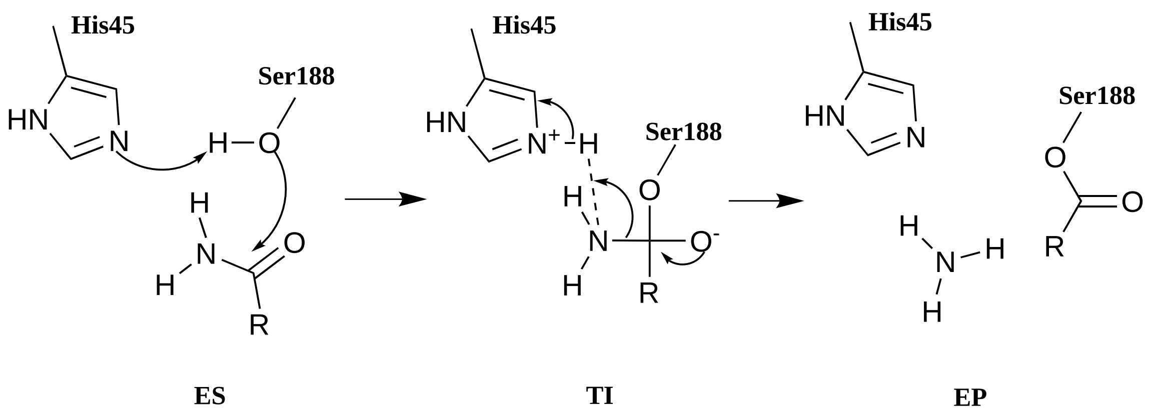


**Fig. S2** Reaction mechanism of the acylation step of the amidase activity of PPE.

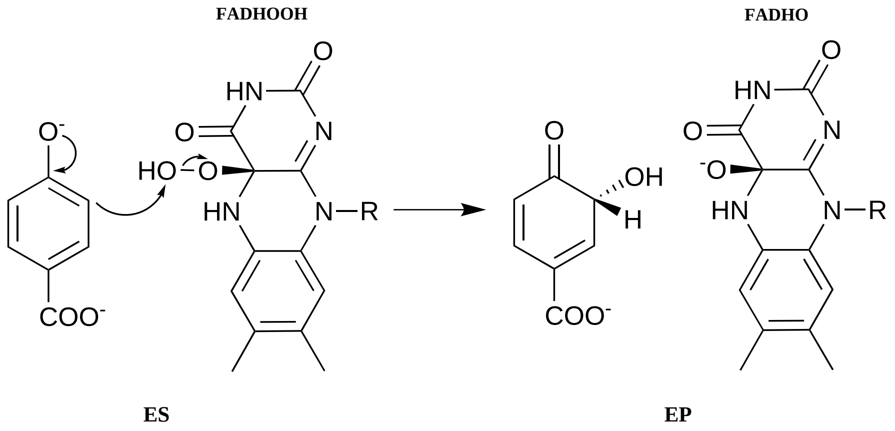


**Fig. S3** Reaction mechanism of the OH-transfer step of PHBH.

the potential of mean force (PMF) by eliminating the part of the harmonic umbrella potential.

# 3 MD simulation

After docking substrate PAPA to wild-type PPE, *p*HB to wild-type PHBH, we gained the stable conformation of PPE-PAPA and PHBH-*p*HB complexes. When the complexes reached dynamical equilibration, the structures were shown in Fig. S4B and Fig. S5B, respectively. In PPE systems, there is a long and narrow substrate binding pocket around the active site to bind polypeptides with different lengths and types of amino acids.[30] For substrate PAPA, which can be catalyzed dominantly by PPE through amidase activity, its C-terminal part is located close to the catalytic triad

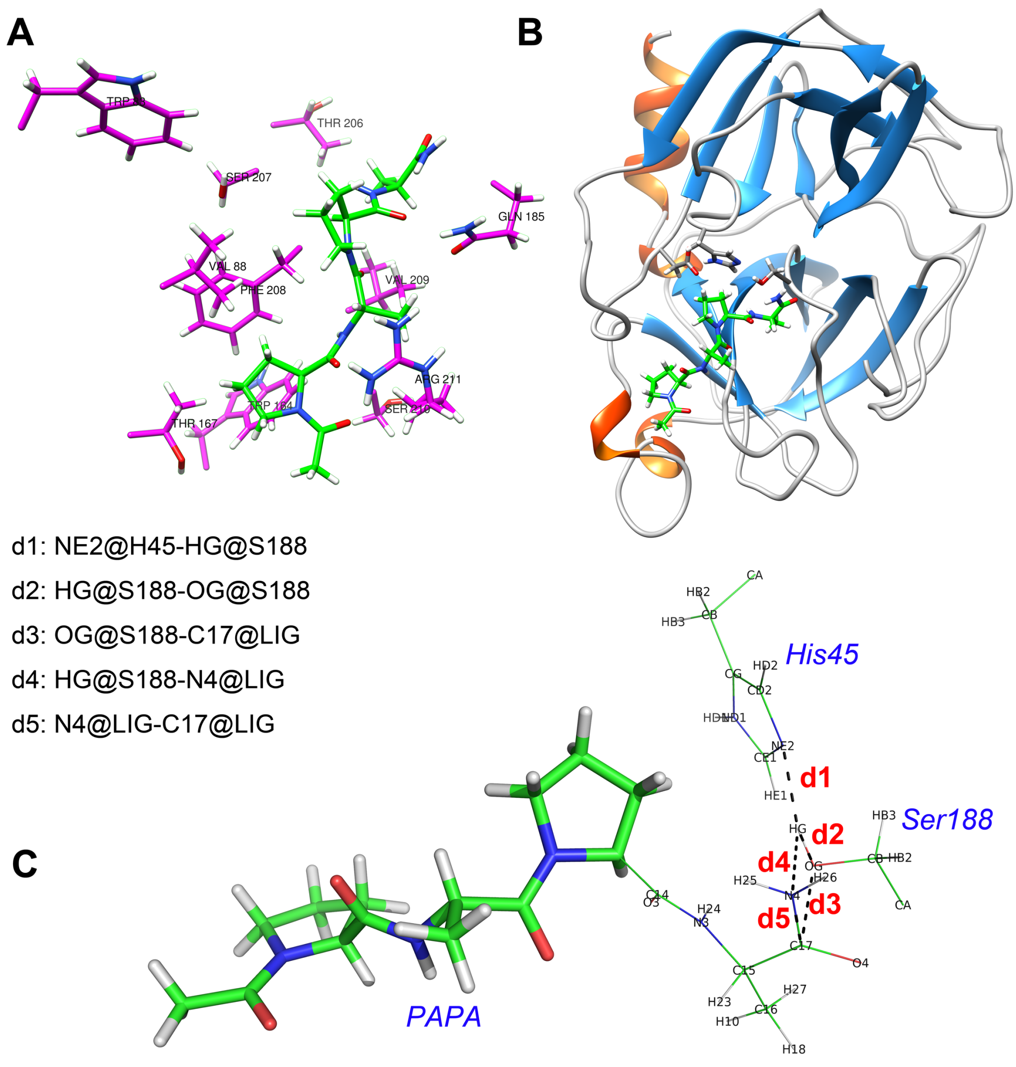


**Fig. S4** (A) Locations of all the 11 mutation sites (colored as pink) in this study. (B) The overall structure of PPE-PAPA complex. The catalytic triad (His45-Asp93-Ser188) is shown in gray sticks. (C) The substrate PAPA and two catalytic residues involved in the forming-breaking of the covalent bonds. The QM part is shown in lines and the MM part of PAPA is shown in sticks. The name of atoms in QM part, and the distances included in reaction coordinates calculations (black dashed lines) of the two steps are labeled in this figure.

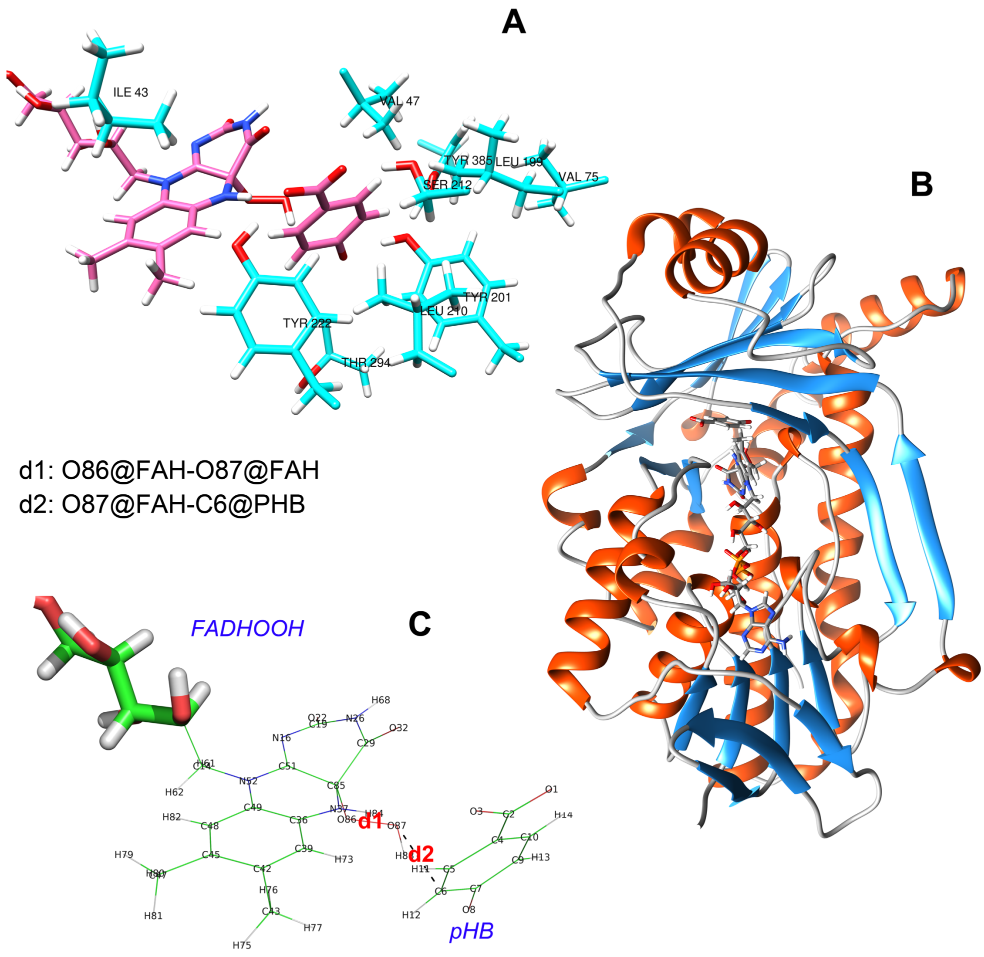


**Fig. S5** (A) Locations of all the 10 mutation sites (colored as cyan) in this study. (B) The overall structure of PHBH-*p*HB complex with the co-factor FADHOOH. (C) The substrate *p*HB and co-factor FADHOOH involved in the forming-breaking of the covalent bonds. The QM part is shown in lines and the MM part of PAPA is shown in sticks. The name of atoms in QM part, and the distances included in reaction coordinates calculations (black dashed lines) are labeled in this figure.

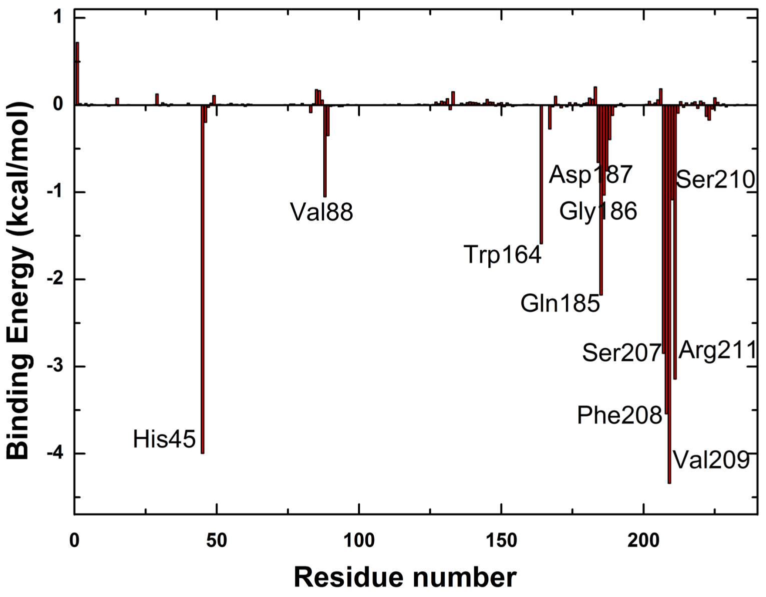


**Fig. S6** The binding free energy ($\Delta G$) decomposition between the ligand PAPA and wild-type PPE. Important residues for ligand binding with $\Delta G <$ -1.0 kcal/mol are labeled in this figure.

of PPE, His45-Asp93-Ser188. Aiming to find out which residues of PPE contribute to the binding of substrate, binding energy decomposition was performed to determine the mutation sites. As illustrated in Fig. S6, residues His46, Val88, Trp164, Gln185, Gly186, Asp187, Ser207, Phe208, Val209, Ser210, and Arg211 are crucial to PAPA binding, especially the residues His46, Phe208, Val209, and Arg211. PAPA has hydrogen bonds with Gln185, Gly186, Ser188, Ser207, Val209, and Arg211, which are helpful to form stable enzyme-substrate complex.

To design the mutation sites for specificity study, the residues for mutation should be located in the active site (have contribution to PAPA binding), and do not affect the reaction mechanism of PAPA deamination (all the enzymes should catalyze substrate PAPA with the same reaction mechanism). Therefore, the mutation sites: 1) may have vdW interactions or H-bonds with substrate; 2) should not be the catalytic residues (His45 and Ser188 are excluded); 3) should have larger side chain than Ala (Gly and Ala are excluded). As a result, mutations W83A, V88A, W164A, T167A, Q185A, T206A, S207A, F208A, V209A, S210A, and R211A are selected to model the mutated systems. The locations of these residues are shown in Fig. S4A. After MD runs of the mutation models, alanine scanning method was applied to calculate the binding energy of each mutated system. The results are listed in Tab. S1. The

binding affinities of W164A, F208A, and R211A decrease significantly from that of wild-type; binding affinities of W83A, T167A, T206A, S207A, and S210A are similar to that of wild-type. Consequently, the van der Waals interactions between Trp164 and PAPA, Phe208 and PAPA, the electrostatic interactions between Arg211 and PAPA have played an important role in determining the binding affinity of mutants W164A, F208A, and R211A.

However, the native binding pocket of PHBH is much smaller than PPE. And there is no catalytic residues in PHBH. Therefore, we selected the residues in the active site, which were around the substrate *p*HB, to make mutations. As shown in Fig. S5A, the mutations I43A, V47A, V75A, L199A, Y201A, L210A, S212A, Y222A, T294A, Y385A were selected for the mutated systems. The results of binding free energy of wild-type and mutated PHBH are listed in Tab. S2. The binding affinities of V47A, Y201A, L210A, S212A, and Y222A decrease significantly from that of wild-type; binding affinities of I43A, V75A, L199A, T294A, and Y385A are similar to that of wild-type. Among them, Y201A, S212A, and Y222A have hydrogen bonds with *p*HB, which will contribute a lot to the binding affinity.

## 4 The reaction mechanism of PPE-PAPA and PHBH-*p*HB complexes

QM/MM MD simulations were performed to obtain the reaction mechanisms of all the wild-type and 21 mutants systems. For simplicity, here we only analyze the reaction mechanism of wild-type complex in detail. For PPE system, there is an obvious tetrahedral intermediate (TI) in the reaction, which divides the whole reaction into two separate steps. As shown in Fig. S7A, in step 1 (from ES to TI), the His45 gets protonated and Ser188 deprotonated, then Ser188 forms a covalent bond with C17 of PAPA; in step 2 (from TI to PS), the amino group of PAPA gets a hydrogen atom from His45 (the HG of Ser188) and is released from PAPA. Backbone nitrogen atoms of Ser188 and Gly186 form two hydrogen bonds with PAPA during the reaction (see Fig. S7A), helping to stabilize the transition state. These two residues compose an oxyanion hole, which is suggested to stabilize the two proposed tetra-

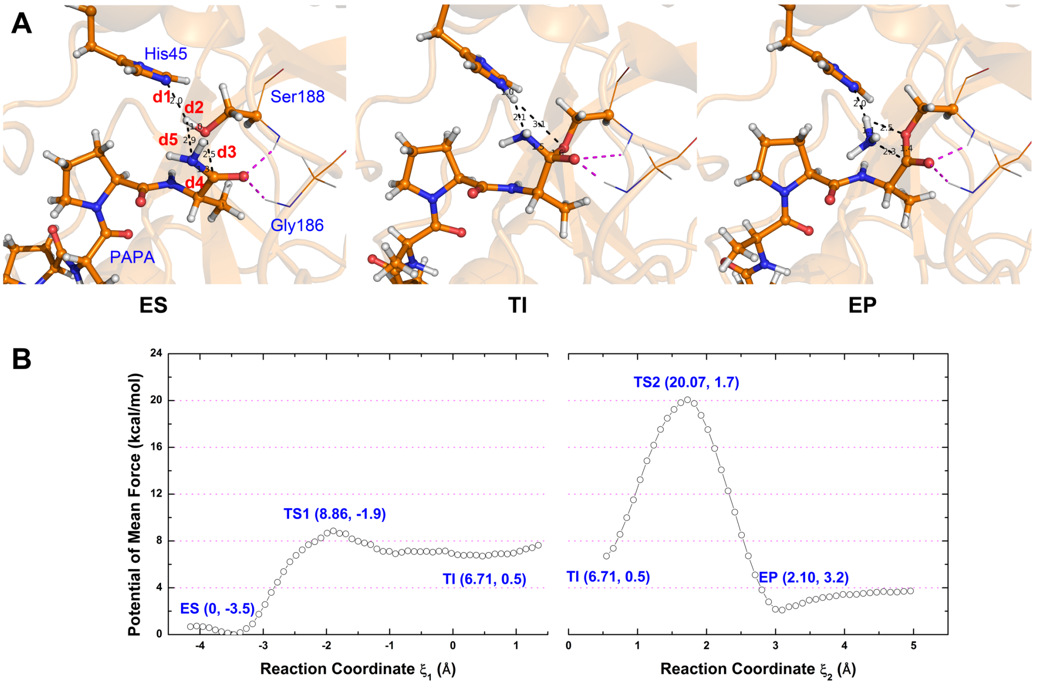


**Fig. S7** (A) The structural characteristics of enzyme-substrate (ES), tetrahedral intermediate (TI), and enzyme-product (EP) complexes in the acylation reaction of amidase activity of PPE. Reaction coordinates (d1, d2, d3, d4, and d5) and their values in different states are labeled. Side chains of catalytic residues His45 and Ser188 are drawn in sticks. Main chains of Ser188 and Gly186 are shown in lines, the hydrogen bonds between them and PAPA are shown in pink dashed lines. (B) Potential of mean force curve of the two steps reaction. The reaction coordinates and energies with respect to the initial state of some important states are labeled.

hedral intermediates on the catalytic pathway. Our findings are consistent with the previous experimental data of Wilmouth *et al.*[31] and Hedstrom *et al*[30].

For PHBH system, the OH-transfer catalysis is one-step reaction. The reaction mechanism is illustrated in Fig. S8. During the reaction, the hydroxyl group is transferred from co-factor FADHOOH to substrate *p*HB, with the O86-O87 bond broken and O87-C6 bond formed. As depicted in Fig. S8B, there are several important hydrogen bonds between *p*HB and the hydroxyl group of Tyr201, Ser212, and Tyr222, which contribute to the stability of the transition state. Moreover, the three mutations, Y201A, S212A, and Y222A, have much lower binding affinity with *p*HB than wild-type enzyme. Therefore, these mutations have a large impact on their activity.

Overall, we constructed the whole free energy curve for the amidase reaction of PPE-PAPA complex (Fig. S7B) and PHBH-*p*HB complex (Fig. S8A) by using WHAM. In PPE system, there is a small barrier (8.86 kcal/mol) in the first step. Then the energy of system drops to 6.71 kcal/mol at the end of the first step. The

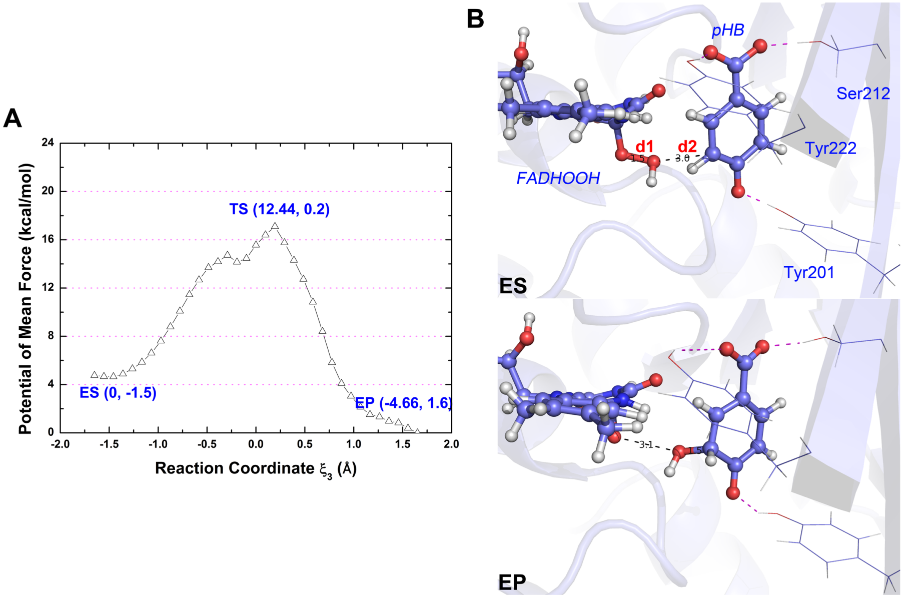


**Fig. S8** (A) Potential of mean force curve of one-step reaction of PHBH. The reaction coordinates and energies with respect to the initial state of some important states are labeled. (B) The structural characteristics of enzyme-substrate (ES) and enzyme-product (EP) complexes in the OH-transfer reaction of PHBH. Reaction coordinates (d1, d2) and their values in different states are labeled. Side chains of Tyr201, Ser212, and Tyr222 are drawn in sticks, the hydrogen bonds between them and *p*HB are shown in pink dashed lines.

rate-limiting step is the second step (from TI to EP). This step needs to overcome an energy barrier of 20.07 kcal/mol, and reaches the EP state at a low energy level of 2.10 kcal/mol. In PHBH system, there is only one barrier on the reaction coordinate, which is 12.44 kcal/mol. The EP state has lower energy of -4.66 kcal/mol than ES state.

Thompson *et al.*[32] reported the $k_{cat}$ value of amidase activity of PAPA is 9.3 /s. According to the transition state theory (TST)[33–37], the estimated energy barrier of this reaction is about 16.72 kcal/mol (300K). And the activation energy of *p*HB was estimated to about 49 kJ/mol (11.71 kcal/mol) in experiments at pH 8.0.[38] In addition, the calculated barrier of PHBH is consistent with the previous QM/MM simulations[39–43]. From this point of view, our QM/MM simulation methods are reasonable. The reaction mechanisms of the selected mutated systems are similar to that of wild-type system. In this study, we only focus on the $k_{cat}$ value (converted form the barrier of the second step in PPE system, and the barrier of OH-transfer step in PHBH system), so we do not analyze the detail reaction mechanism of each mutated system here. Mutant W83A has the highest reaction barrier of all PPE systems, and

Mutant Y385A has the highest reaction barrier of all PHBH systems. The barriers of V88A, F208A, and V209A are slightly lower than that of wild-type PPE, and that barrier of S212A is a bit lower than wild-type PHBH. Of all the 12 PPE systems, wild-type system has the highest activity. V88A, S207A, V209A, and S210A have slightly lower activity than wild-type. W164A has the lowest activity. Of all the 11 PHBH systems, I43A has the highest activity, which is slightly higher than wild-type. The activities of V47A, V75A, L199A, T294A, and Y385A are similar as that of wild-type.

## References


[1] J. Wang and G. M. Verkhivker, *Physical Review Letters*, 2003, **90**, 188101.

[2] J. Wang, X. Zheng, Y. Yang, D. Drueckhammer, W. Yang, G. Verkhivker and E. Wang, *Physical Review Letters*, 2007, **99**, 198101.

[3] J. D. Bryngelson and P. G. Wolynes, *Proceedings of the National Academy of Sciences*, 1987, **84**, 7524–7528.

[4] J. D. Bryngelson, J. N. Onuchic, N. D. Socci and P. G. Wolynes, *Proteins: Structure, Function, and Bioinformatics*, 1995, **21**, 167–195.

[5] J. Janin, *Proteins: Structure, Function, and Bioinformatics*, 1996, **25**, 438–445.

[6] P. A. Rejto and G. M. Verkhivker, *Proceedings of the National Academy of Sciences*, 1996, **93**, 8945–8950.

[7] C.-J. Tsai, S. Kumar, B. Ma and R. Nussinov, *Protein Science*, 1999, **8**, 1181–1190.

[8] B. N. Dominy and E. I. Shakhnovich, *Journal of Medicinal Chemistry*, 2004, **47**, 4538–4558.

[9] Z. Liu, B. N. Dominy and E. I. Shakhnovich, *Journal of the American Chemical Society*, 2004, **126**, 8515–8528.

[10] Y. Levy, P. G. Wolynes and J. N. Onuchic, *Proceedings of the National Academy of Sciences of the United States of America*, 2004, **101**, 511–516.

[11] J. Wang, *Chemical physics letters*, 2006, **418**, 544–548.

[12] J. D. Bryngelson and P. G. Wolynes, *The Journal of Physical Chemistry*, 1989, **93**, 6902–6915.

[13] J. N. Onuchic, P. G. Wolynes, Z. Luthey-Schulten and N. D. Socci, *Proceedings of the National Academy of Sciences*, 1995, **92**, 3626–3630.

[14] J. N. Onuchic and P. G. Wolynes, *Science*, 1995, **267**, 1619–1620.

[15] J. Wang, L. Xu and E. Wang, *Biophysical Journal*, 2007, **92**, L109–L111.

[16] Z. Yan, X. Zheng, E. Wang and J. Wang, *Chemical Science*, 2013, **4**, 2387–2395.

[17] B. Derrida, *Physical Review B*, 1981, **24**, 2613.

[18] S. S. Plotkin, J. Wang and P. G. Wolynes, *The Journal of chemical physics*, 1997, **106**, 2932–2948.

[19] Z. Yan and J. Wang, *Scientific reports*, 2012, **2**, 309.

[20] V. Kräutler, W. F. van Gunsteren and P. H. Hünenberger, *Journal of Computational Chemistry*, 2001, **22**, 501–508.

[21] T. Darden, D. York and L. Pedersen, *The Journal of chemical physics*, 1993, **98**, 10089–10092.

[22] H. Gohlke, C. Kiel and D. A. Case, *Journal of Molecular Biology*, 2003, **330**, 891–913.

[23] W. C. Still, A. Tempczyk, R. C. Hawley and T. Hendrickson, *Journal of the American Chemical Society*, 1990, **112**, 6127–6129.

[24] A. Onufriev, D. Bashford and D. A. Case, *Proteins: Structure, Function, and Bioinformatics*, 2004, **55**, 383–394.

[25] J. M. Rosenbergl, *Journal of Computational Chemistry*, 1992, **13**, 1011–1021.

[26] J. Kästner, *Wiley Interdisciplinary Reviews: Computational Molecular Science*, 2011, **1**, 932–942.

[27] M. Elstner, D. Porezag, G. Jungnickel, J. Elsner, M. Haugk, T. Frauenheim, S. Suhai and G. Seifert, *Physical Review B*, 1998, **58**, 7260.

[28] T. Krüger, M. Elstner, P. Schiffels and T. Frauenheim, *The Journal of chemical physics*, 2005, **122**, 114110.

[29] A. Grossfield, *WHAM: the weighted histogram analysis method*, http://membrane.urmc.rochester.edu/content/wham, 2012.

[30] L. Hedstrom, *Chemical Reviews*, 2002, **102**, 4501–4524.

[31] R. C. Wilmouth, I. J. Clifton, C. V. Robinson, P. L. Roach, R. T. Aplin, N. J. Westwood, J. Hajdu and C. J. Schofield, *Nature Structural & Molecular Biology*, 1997, **4**, 456–462.

[32] R. C. Thompson and E. R. Blout, *Proceedings of the National Academy of Sciences*, 1970, **67**, 1734–1740.

[33] H. Eyring, *The Journal of Chemical Physics*, 1935, **3**, 107–115.

[34] P. Pechukas, *Annual Review of Physical Chemistry*, 1981, **32**, 159–177.

[35] D. G. Truhlar, B. C. Garrett and S. J. Klippenstein, *The Journal of physical chemistry*, 1996, **100**, 12771–12800.

[36] K. J. Laidler and M. C. King, *The Journal of physical chemistry*, 1983, **87**, 2657–2664.

[37] E. V. Anslyn and D. A. Dougherty, *Modern physical organic chemistry*, University Science Books, 2006.

[38] W. J. BERKEL and F. MÜLLER, *European Journal of Biochemistry*, 1989, **179**, 307–314.

[39] J. Kästner, H. M. Senn, S. Thiel, N. Otte and W. Thiel, *Journal of Chemical Theory and Computation*, 2006, **2**, 452–461.

[40] H. M. Senn, S. Thiel and W. Thiel, *Journal of chemical theory and computation*, 2005, **1**, 494–505.

[41] F. Claeyssens, J. N. Harvey, F. R. Manby, R. A. Mata, A. J. Mulholland, K. E. Ranaghan, M. Schütz, S. Thiel, W. Thiel and H.-J. Werner, *Angewandte Chemie*, 2006, **118**, 7010–7013.

[42] L. Ridder, B. A. Palfey, J. Vervoort and I. M. Rietjens, *FEBS letters*, 2000, **478**, 197–201.

[43] L. Ridder, J. N. Harvey, I. M. Rietjens, J. Vervoort and A. J. Mulholland, *The Journal of Physical Chemistry B*, 2003, **107**, 2118–2126.

**Tab. S1** Binding energy ($\Delta G_{bind}$, kcal/mol), reaction barrier ($\Delta G^{\ddagger}$, kcal/mol), activity ($ln(k_{cat}/K_m)$), and ISR values of wild-type and mutated PPE systems.

| | $\Delta G_{bind}$ (kcal/mol) | $\Delta G^{\ddagger}$ (kcal/mol) | $ln(k_{cat}/K_m)$ | ISR |
|---|---|---|---|---|
| wt | -44.956 | 20.066 | 72.204 | 4.739 |
| W83A | -44.551 | 23.155 | 66.342 | 3.503 |
| V88A | -43.444 | 19.768 | 70.233 | 3.880 |
| W164A | -41.028 | 21.193 | 63.724 | 2.985 |
| T167A | -44.605 | 22.632 | 67.313 | 3.265 |
| Q185A | -43.422 | 22.116 | 66.191 | 3.248 |
| T206A | -44.994 | 22.838 | 67.617 | 3.874 |
| S207A | -44.104 | 20.470 | 70.096 | 5.575 |
| F208A | -41.993 | 19.588 | 68.034 | 3.827 |
| V209A | -43.821 | 19.387 | 71.439 | 5.112 |
| S210A | -44.779 | 20.346 | 71.436 | 4.160 |
| R211A | -41.816 | 21.532 | 64.477 | 3.386 |

**Tab. S2** Binding energy ($\Delta G_{bind}$, kcal/mol), reaction barrier ($\Delta G^{\ddagger}$, kcal/mol), activity ($ln(k_{cat}/K_m)$), and ISR values of wild-type and mutated PHBH systems.

| | $\Delta G_{bind}$ (kcal/mol) | $\Delta G^{\ddagger}$ (kcal/mol) | $ln(k_{cat}/K_m)$ | ISR |
|---|---|---|---|---|
| wt | -57.4961 | 12.440 | 106.041141 | 5.15502 |
| I43A | -58.2859 | 12.086 | 107.959946 | 5.43313 |
| V47A | -54.6325 | 12.144 | 101.733810 | 5.72317 |
| V75A | -57.9432 | 13.152 | 105.596752 | 5.51125 |
| L199A | -56.2255 | 12.744 | 103.399637 | 4.06623 |
| Y201A | -51.3144 | 12.547 | 95.491401 | 2.54413 |
| L210A | -53.3688 | 13.498 | 97.342432 | 4.35560 |
| S212A | -51.8579 | 10.582 | 99.699587 | 4.13121 |
| Y222A | -51.6133 | 13.506 | 89.351323 | 3.63056 |
| T294A | -56.6646 | 12.143 | 105.144479 | 5.14388 |
| Y385A | -58.4105 | 14.490 | 104.136091 | 5.29454 |

**Tab. S3** Distance (Å) and mulliken charge (e) of all important states in the acylation reaction of PPE. ES, TS1, TI, TS2, and EP states correspond to the states in Fig. S7B.

| | | ES | TS1 | TI | TS2 | EP |
|---|---|---|---|---|---|---|
| distance (Å) | d1 | 1.991 | 1.196 | 1.031 | 1.244 | 1.981 |
| | d2 | 0.958 | 1.401 | 3.083 | 2.884 | 2.532 |
| | d3 | 2.518 | 2.219 | 1.583 | 1.360 | 1.435 |
| | d4 | 2.883 | 2.980 | 2.091 | 1.362 | 1.054 |
| | d5 | 1.346 | 1.380 | 1.456 | 1.875 | 2.277 |
| charge (e) | NE2 | -0.389 | -0.110 | 0.013 | -0.180 | -0.368 |
| | HG | 0.327 | 0.280 | 0.234 | 0.238 | 0.210 |
| | OG | -0.470 | -0.700 | -0.425 | -0.461 | -0.273 |
| | C17 | 0.530 | 0.540 | 0.601 | 0.608 | 0.630 |
| | N4 | -0.322 | -0.320 | -0.511 | -0.441 | -0.474 |

**Tab. S4** Distance (Å) and mulliken charge (e) of all important states in OH-transfer reaction of PHBH. ES, TS, and EP states correspond to the states in Fig. S8A.

| | | ES | TS | EP |
|---|---|---|---|---|
| distance (Å) | d1 | 1.477 | 2.142 | 3.133 |
| | d2 | 2.967 | 1.942 | 1.482 |
| charge (e) | O86 | -0.238 | -0.659 | -0.951 |
| | O87 | -0.332 | -0.440 | -0.474 |
| | C6 | -0.229 | -0.011 | 0.171 |

**Tab. S5** The computational time (unit in days) of PPE and PHBH systems. Each MD run (normal MD and QM/MM MD simulations) is performed with 12 cpu in parallel.

| | PPE (12 systems) | PHBH (11 systems) |
|---|---|---|
| normal MD | ∼ 3 per sys | ∼ 5 per sys |
| free energy calculation | ∼ 1 per sys | ∼ 1 per sys |
| QM/MM parameterization | ∼ 15 | ∼ 10 |
| QM/MM run | ∼ 7 per sys | ∼ 4 per sys |
| ISR calculation | ∼ 4 per sys | ∼ 4 per sys |
